# Sustainability indicators for gastrointestinal endoscopy: a framework proposed by the Canadian Association of Gastroenterology and the American Society for Gastrointestinal Endoscopy

**DOI:** 10.1093/jcag/gwag030

**Published:** 2026-08-03

**Authors:** Desmond Leddin, John Igoe, Geoffrey C Nguyen, Sonali Palchaudhuri, Neal Shahidi, Heiko Pohl

**Affiliations:** Department of Medicine, Dalhousie University, Halifax, Nova Scotia, B3H 4H6, Canada; Department of Medicine, Dalhousie University, Halifax, Nova Scotia, B3H 4H6, Canada; Mount Sinai Inflammatory Bowel Disease Center, University of Toronto, Toronto, Ontario, M5G 1X5, Canada; Department of Gastroenterology, Massachusetts General Hospital, Boston, Massachusetts, 02114, United States; Department of Medicine, University of British Columbia, Vancouver, British Columbia, V6T 1Z4, Canada; Department of Gastroenterology and Hepatology, VA Medical Center, White River Junction, Vermont, 05009, United States; Dartmouth Hitchcock Medical Center, Lebanon, New Hampshire, 03756, United States

## Introduction

Globally, climate change is a major threat to public health.^1^ Paradoxically, the health care sector, including GI endoscopy, contributes to the problem by generating greenhouse gases, pollution, and waste.^2–4^ Recognizing this, GI societies have committed to supporting endoscopy teams in transitioning to a sustainable model of care that delivers maximal quality with minimal environmental impact.^5^^,^^6^ Sustainability has also been proposed as an additional quality domain, alongside effectiveness, safety, timeliness, efficiency, patient experience, and equity.^7^

Recent studies have examined waste and carbon emissions generated by performing endoscopic procedures. On average, 1 endoscopic procedure in the United States generates between 2 and 3 kg of waste, most of which is sent to landfill.^8^^,^^9^ With respect to carbon emissions, performing a single endoscopic procedure generates between 28 and 55 kgCO_2_e.^10–12^ For reference, it takes 1 fully grown tree an entire year to offset about 20 kgCO_2_e. Other environmental impacts, such as contributions to pollution, plastic waste, and biodiversity, have not been examined. Although recommendations for transitioning to more sustainable endoscopy practices have been published,^13–15^ endoscopy teams may find it challenging to determine which interventions are most relevant, impactful, and achievable. This document introduces a set of objective sustainability indicators and proposes actions to achieve them. It is designed to assist endoscopy units in planning and assessing their progress toward sustainability and may also serve as a framework for integrating sustainability into existing recognition programs, such as the ASGE Endoscopy Unit Recognition Program.

It is important to note that sustainability should be viewed as a path rather than a destination, which affords opportunities for continuous improvement. Indeed, practicing sustainability is not only about minimizing waste and lowering the carbon footprint, but also about aligning endoscopic practices with a broader cultural shift. Small-scale changes, such as those within an individual endoscopy unit, may in turn inspire system-wide improvements.^16^^,^^17^

## Objectives

The objective of this joint project between the Canadian Association of Gastroenterology (CAG) and the American Society for Gastrointestinal Endoscopy (ASGE) was to establish a set of indicators to guide endoscopy units in transitioning toward sustainable practices.

## Methods

Representatives of the ASGE Taskforce for Sustainable Endoscopy, the ASGE Quality Assurance in Endoscopy Committee, and the CAG Climate and Quality Affairs Committee prepared the initial draft of the document. Meetings were held virtually.

Although evidence for sustainable practices in GI endoscopy is emerging, its absence is not unique to sustainability, and issuing guidance statements can still be valuable (eg, as done during the COVID-19 pandemic). We anticipate that the actions outlined in this document will be modified as evidence accumulates.

### Categories, indicators, actions, targets, and aspirational goals

Indicator categories within endoscopy practice were identified through an iterative process and interactive discussion. The choices were informed by existing literature on emissions and waste associated with endoscopy. Seven categories of GI endoscopy practice were selected: administration, education, appropriateness of procedures, consumables, waste, travel, and energy (Figure 1 and Table 1).

**Figure 1:**
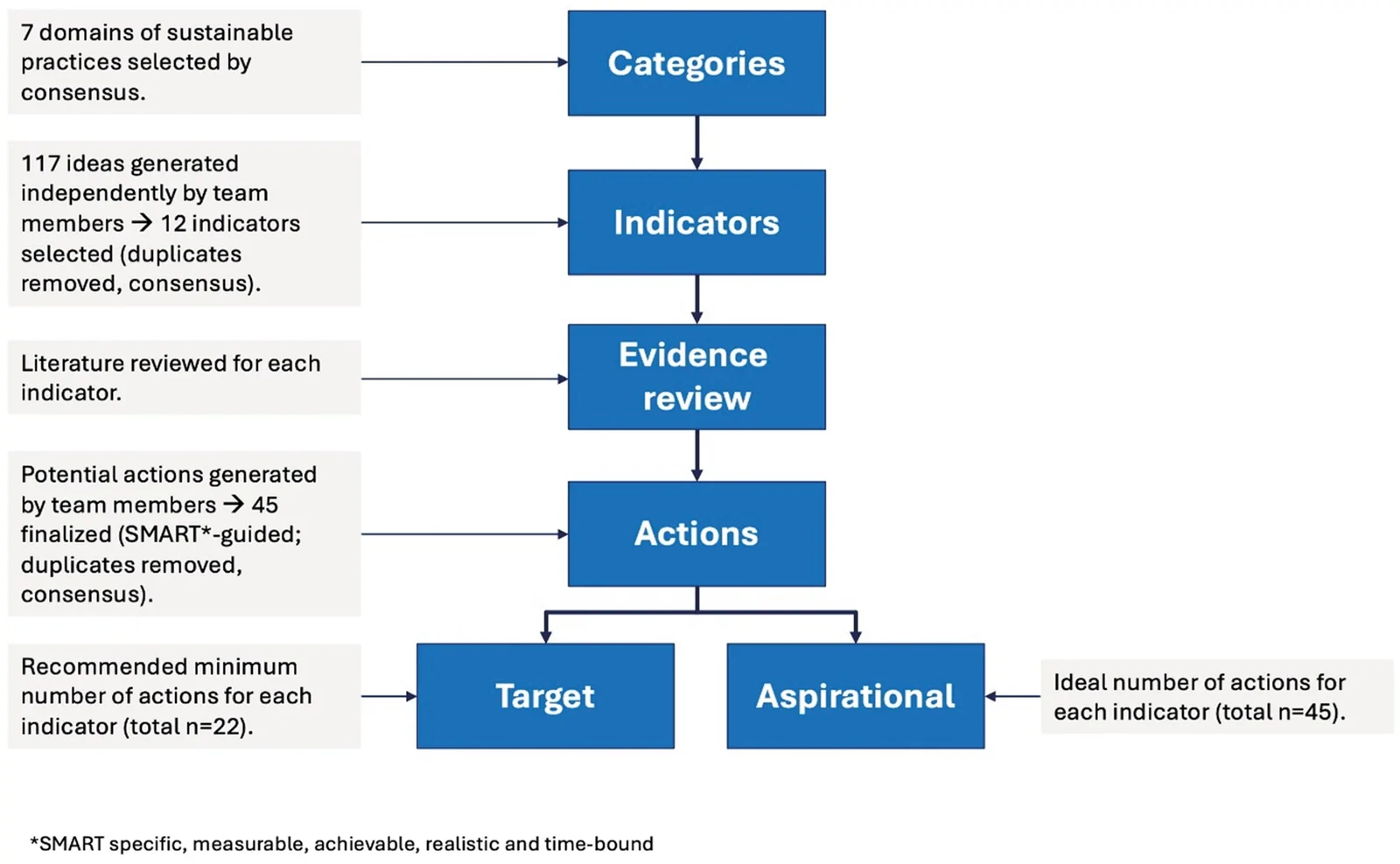
Development of sustainability categories, indicators, targets, and aspirational goals.

**Table 1:** Sustainability indicators within 7 categories of endoscopy practice and proposed actions for each indicator.

Sustainability indicator	Type of indicator	Proposed actions for each indicator
**Administration**		
1. Establish an administrative structure to support environmental sustainability	Structural	Appoint a sustainability lead. Establish a “Green Team.” Include environmental sustainability as a standing item on unit management meetings. *Target:* complete at least 2 actions. *Aspirational:* complete all actions.
2. Establish an environmental sustainability plan for the endoscopy unit, including goals, actions, and mechanisms for monitoring progress.	Structural	Recommended content of a sustainability plan: Define the aims and scope of carbon emission reduction. Include education initiatives. Address reduction/optimization of patient and staff travel. Outline management of consumables. Outline management of waste. Plan to reduce unit energy use. Plan to adapt to climate change and to build resilience. Plan to monitor practice changes and their environmental and financial impact. *Target:* incorporate at least 4 content items. *Aspirational:* incorporate all items.
**Education**		
3. Educate all members of the endoscopy team on topics related to green endoscopy.	Process	Recommended events and topics: Include environmental sustainability as a topic in rounds, journal clubs, and continuing education (at least once per year). Educate staff and patients on the contribution of travel to endoscopy-related emissions and strategies to reduce it (once per year). Educate staff on management of consumables (once per year). Educate staff on waste management (once per year). Educate staff on energy conservation (once per year). Develop and distribute educational material for patients. Display information material (eg, posters) in staff and patient areas outlining the unit’s sustainability initiatives. *Target:* complete at least 4 actions. *Aspirational:* complete all actions.
4. Implement point-of-care decision-making support tools.	Process	Label bins clearly to promote correct disposal of waste. Establish a reminder system to ensure printers, monitors, and other electronic devices are turned off when not in use. *Target:* complete at least 1 action. *Aspirational:* complete both actions.
**Appropriateness of procedures**		
5. Minimize inappropriate endoscopic procedures.	Process	Implement a process to support adherence to best practice guidelines (eg, minimize procedures with unsupported indication).* Establish a process to encourage low-waste, low-carbon alternatives to endoscopy.* Improve utilization of available procedure time slots.* *Target:* complete at least 1 action. *Aspirational:* complete all actions.
**Consumables**		
6. Reduce consumables.	Process	Educate endoscopists and instrument technicians on the optimal use of instruments (eg, use of snares for all polyps; see text for additional examples). Assess and report the number of snares and forceps used per colonoscopy by individual endoscopists at 2 time points (eg, 3 months apart). Consolidate the endoscopy unit’s inventory. Minimize expired instruments and supplies.* Minimize paper use.* *Target:* complete at least 2 actions. *Aspirational:* complete all actions.
7. Reuse consumables.	Process	Review instruments and supplies and replace single-use items with reusable alternatives.* *Target:* complete this action.
**Waste**		
8. Optimize waste disposal.	Process	Conduct a waste audit to identify erroneous disposal practices. Reduce the volume of hazardous waste disposed of incorrectly.* Institute a recycling program that includes recyclable items (as permitted under local contract) and monitor progress. *Target:* complete at least 1 action. *Aspirational:* complete all actions.
**Travel**		
9. Perform telehealth visits when feasible, clinically appropriate, and preferred by the patient.	Process	Review the frequency of preprocedure and postprocedure visits that could be conducted virtually. Increase the number of virtual care visits, as appropriate.* Offer patients the option to have a virtual visit when clinically appropriate. Incorporate virtual care options and their rationale into unit written materials and discharge protocols. *Target:* complete at least 2 actions. *Aspirational:* complete all actions.
10. Maximize double procedures on the same days for patients requiring more than one procedure.	Process	Review cases in which patients had 2 procedures on separate days but could have undergone both on the same day, and increase the proportion of such double procedures.* *Target:* complete this action.
**Energy**		
11. Measure the endoscopy unit’s energy use.	Process	Establish a baseline of electricity use in the endoscopy unit (including lighting, computers, monitors, processors, washers). Establish a baseline for HVAC use. *Target:* complete at least 1 action. *Aspirational:* complete both actions.
12. Reduce the endoscopy unit’s energy use.	Process	Replace incandescent lighting with LED lights. Install motion-activated light switches with automatic shut-off. Turn off endoscopy equipment between cases. Turn off computers, printers, and monitors when not in use (eg, at the end of the day). Check and adjust HVAC setpoints to minimize energy demand (see text). Assess air exchange rates and reduce them when the room is not in use. *Target:* complete at least 3 actions. *Aspirational:* complete all actions.

* Endoscopy units need to benchmark their current performance (through reviewing their current practice) and set a goal relative to the benchmark (eg, 50% improvement). The action item is completed if the endoscopy unit’s individual goal is reached.

Abbreviations: HVAC, Heating, ventilation, and air-conditioning; LED, light-emitting diode.

Within these categories, statements were formulated to represent sustainability indicators following these principles: (1) statements should be clear; (2) indicators should be practical, achievable, and measurable; and (3) indicators should be relevant and impactful.^18^ As the first step, each member of the team independently listed their suggestions. Initially, 117 individual indicators were considered. These were collated, duplicates eliminated, and similar items merged. Indicator suggestions were then ranked independently by each working group member. The suggested indicators and their ranking were discussed, and 12 final indicators were selected using the criteria outlined earlier. For each indicator, an additional literature review was conducted. A rationale for each indicator is provided, along with a summary of supporting evidence and reasoning where data are lacking.

Each sustainability indicator was then aligned with proposed action items required to achieve it. Drawing on published literature, each team member independently generated a list of potential actions for each indicator. The final list was selected by consensus and guided by the principle that each action should meet specific, measurable, achievable, realistic, time-bound criteria (ie, achievable within a given time).

For each indicator, both a minimum target and an aspirational goal were specified. Aspirational goals represent the ideal scenario. Recognizing that units differ in their current status (some may have already implemented changes) and in the resources available to them, we acknowledge that not every unit will be able to achieve all actions. Therefore, minimum achievable targets were determined for each indicator, taking into account difficulty and feasibility, and were finalized by consensus of the working group. Importantly, these targets and aspirational goals may also serve as a framework for future unit recognition programs.

## External Review

The document was reviewed by the *Gastrointestinal Endoscopy* Editorial Board, Sustainable Endoscopy Task Force, and ASGE Governing Board and made available for public comment on the ASGE website for 31 days between December 17, 2024, and January 17, 2025.

## Sustainability Indicators

### Administration

1. *Establish an administrative structure to support environmental sustainability.*

Incorporation of sustainability into endoscopy unit operations represents a significant change in practice.^19^ It requires administrative support to initiate projects, expand the team, secure buy-in from members, reassess changes, and make adjustments.

A sustainability lead can help drive initiatives, support organizational changes, and ensure that plans are executed.^20^^,^^21^ Importantly, leads serve as the “face” of sustainable efforts and may inspire cultural change.^22^^,^^23^ Although transformation may begin with a lead, long-term success requires leadership support and the involvement of stakeholders such as procurement, infection control, facility management, and quality assurance.^24^ The lead may work individually within the management team or as part of a Green Team.^25^ One way to promote sustainability is to list it as a perspective in unit business meetings. Rather than as a stand-alone item, it can become one of the lenses through which management decisions are made.

2. *Develop an environmental sustainability plan for the endoscopy unit, including goals, actions, and mechanisms for monitoring progress.*

A unit’s sustainability plan can provide a roadmap for progress toward sustainable practice.^26^^,^^27^ In developing such a plan, all team members may participate in defining objectives, setting milestones, and agreeing on initiatives and practical steps. The plan may include goals to reduce carbon emissions and waste, such as a commitment to carbon neutrality over time, or a percentage reduction from baseline. Table 1 outlines recommended content. Action items may be implemented as quality improvement initiatives, and additional actions not addressed in the following sections can also be included. Examples include strategies to adapt to climate change and build resilience against threats to infrastructure and supply chains.^28^ Finally, the plan should address how to monitor any practice changes and evaluate both environmental and financial effects.

### Education

3. *Educate all members of the endoscopy team on topics related to green endoscopy.*

To support decision-making around the unit’s sustainability plan and actions, team-based education is essential. A lack of knowledge has been identified as a barrier to implementing sustainable practices.^29^^,^^30^ Educational efforts are most effective when team members have some control over actions and when they work within a supportive organizational culture.^17^

Education also fosters engagement. For example, training on waste disposal and the development of clear recycling guidelines can improve waste management and contribute to energy savings.^17^^,^^31^ The “team” in this context includes not only health care providers such as nurses and physicians but also administrative staff, cleaning and waste management personnel, facility maintenance staff, and information technology support. The focus of educational efforts can vary by role. For physicians, avoiding unnecessary disposable instruments is most relevant, whereas administrative staff may benefit more from training on energy efficiency and sustainable procurement.^10–12^ Education can be integrated into existing formats such as grand rounds, journal clubs, and continuing education presentations.

4. *Implement point-of-care decision-making support tools.*

Education can be facilitated through point-of-care decision-making tools. Educational interventions―such as targeted signage over bins and other point-of-care prompts―have reduced waste and improved recycling rates by 17% to 54%.^32^^,^^33^ Similar tools, such as checklists, can extend beyond waste reduction by helping staff consistently shut down electronic equipment at the end of the day and avoid unnecessary energy consumption.^34^

Patient engagement is another important aspect of transitioning to sustainable practice. Patients need to be assured that quality will not be compromised and may even be enhanced. Their support has broader societal and cultural dimensions. Educating patients and the public on the links between environmental change and health can promote understanding of practice changes and support informed choices.^35^ This can be achieved through posters and signage in endoscopy units, as well as through materials distributed before procedures and at discharge. Examples include information on the benefits of virtual follow-up visits, the impact of travel, and the rationale for same-day procedures, along with links to resources on climate and health.

### Appropriateness of procedures

5. *Minimize inappropriate endoscopic procedures.*

Inappropriate or unnecessary performance of colonoscopy and upper endoscopy has been reported in 20% to 50% of cases.^36^^,^^37^ This practice not only affects resources and access to care but also increases environmental impact. A key strategy to reduce the environmental impact of endoscopy, therefore, is to minimize procedures and interventions that are not indicated or are of low value―in other words, to adopt a “less is more” approach.^38^ Notably, closer adherence to guidelines may reflect not only high-quality care but also best sustainable practice. It should, however, be noted that not all guidance is based on strong evidence, and some guidelines may favor performing procedures rather than omitting them.^36^ Incorporating a sustainability perspective when developing guidelines is increasingly recognized.

While challenging, reviewing procedure indications for their appropriateness can identify opportunities for practice change and educate team members on best practices. In addition, modifications to electronic medical records, such as prompts regarding the timing of follow-up procedures, may help reduce overutilization.

Consideration should also be given to using appropriate alternatives to endoscopic procedures when able. For example, noninvasive colorectal cancer screening tests represent alternative screening options and may be associated with a lower carbon footprint.^39^^,^^40^ Similarly, intestinal US combined with fecal calprotectin offers a more sustain-able alternative to colonoscopy for monitoring therapeutic response in patients with inflammatory bowel disease, although comparative data on carbon footprint are not yet available.^41^^,^^42^ Decision-support algorithms may assist clinicians in recognizing and choosing more sustainable practice alternatives with comparable efficacy and safety.

Finally, making full use of time and unit space can translate into environmental and financial benefits, because heating, ventilation, and air-conditioning (HVAC) and lighting systems consume energy regardless of whether patients are present. Utilization can be improved by preventing repeat procedures (eg, by ensuring adequate bowel preparation), reducing no-show rates, and maximizing available time slots, for example, by accommodating same-day add-on cases.

### Consumables

6. *Reduce consumables.*

Consumables refer to instruments and supplies used in endoscopy before, during, and after procedures. Historically, many items were cleaned, disinfected, or sterilized and then reused.^43^ Most reusable supplies and instruments have since been replaced by single-use items, largely because of infection control concerns. Not surprisingly, consumables generate substantial waste and contribute up to 21% to the carbon footprint of performing an endoscopy.^11^^,^^12^ Generally, the environmental impact of consumables can be lowered by reducing their use and by adopting reusable items where possible.

Reducing the use of consumables can be achieved in several ways. For example, only the supplies and instruments essential for a given task should be used. A ≤3-mm polyp can be removed with a snare rather than forceps, minimizing the number of tools required if another larger polyp also needs a snare. In 1 study, this practice reduced the number of forceps by about 44 per 100 colonoscopies.^44^ Other green practices include performing cold resection of all polyps <10 mm and avoiding clip closure after resection of polyps <20 mm or after cold resection of any polyp.^45^ Additional practices include forgoing submucosal injection for polyps ≤15 mm and avoiding unnecessary tissue sampling, such as biopsy of an irregular Z-line.^46^^,^^47^ These examples align with current guideline recommendations. In addition, team-based steps, such as communicating with the technician before opening packages, can also help reduce the number of unused instruments.

Furthermore, consolidating and optimizing the inventory of supplies and instruments can reduce overall use. For example, stocking several snares of similar function and quality is often unnecessary and increases the likelihood that unused snares will expire. Regularly reviewing expiration dates and prioritizing the use of items nearing expiration (first-in, first-out principle) are practical steps to minimize waste. Avoiding urgent or overnight orders will also reduce carbon emissions, as such items are often shipped by air and generate greater emissions than larger ground shipments. In addition, items with a lower environmental impact can be prioritized for purchase. Sterile gloves, for instance, have nearly 12 times the environmental footprint of nonsterile gloves.^48^

Switching to paperless systems is another strategy that reduces environmental impact.^49^^,^^50^ Procedure reports and discharge instructions can be provided electronically. However, the second-order effects of digitalization―such as the energy demands of data storage―should also be recognized.^51^

7. *Reuse consumables.*

Reusing items aligns with the principles of a circular economy, which avoids exploiting finite resources for new manufacturing.^52^ However, only a few items used in endoscopy are currently reusable, including water bottles, blood pressure cuffs, electrocardiogram leads, pulse oximeters, endoscopes, scope valves, and bougie dilators. Personal protective equipment may also be reusable, such as washable gowns, designated face masks, and eye shields.

Reusable products require more resources for manufacturing and reprocessing than single-use devices.^53^^,^^54^ Nevertheless, their environmental benefits over a lifetime are well documented.^53^^,^^55–59^ For example, reusable pulse oximeters reach their environmental break-even point after only 2 to 3 uses. Switching to reusables may also lower costs.^60^^,^^61^

The introduction of single-use endoscopes has been controversial. Although they may reduce infection risk, replacing reusable endoscopes with single-use versions would increase net waste by 40%. Similarly, switching to reusable duodenoscopes would raise the carbon footprint 23- to 75-fold.^62^^,^^63^ Any potential upfront benefit from reduced infection risk is outweighed by the indirect harms associated with their environmental impact.^62^

The process of substituting single-use products with reusables can begin with a review of items currently in use. Units can then develop a plan relative to their benchmark, set specific targets for substitution, and monitor progress. Information on available reusables can be obtained from society websites or directly from vendors.^64^

Finally, patients can be engaged by encouraging them to bring their own reusable items, such as socks, cups, or containers for dentures or glasses. Although the environmental benefit is small, involving patients in this way can promote cultural change and reinforce commitment to sustainable endoscopy practice.

### Waste

8. *Optimize waste disposal.*

As a high-volume specialty, endoscopy generates considerable physical waste.^3^^,^^9^^,^^65–67^ It is the third-largest generator of hazardous waste (regulated medical waste) among hospital departments.^67^ A single endoscopic procedure generates 1 to 2 kg of waste in Europe and 2 to 3 kg of waste in the United States.^9^^,^^68^^,^^69^ In the United States, approximately two-thirds of endoscopic waste is disposed of in municipal landfills, and about 30% as hazardous waste.^9^^,^^69^ Up to 30% of waste is potentially recyclable.^8^^,^^9^^,^^65^^,^^69^ Although waste contributes only 3% of the total carbon footprint of endoscopy units, plastic waste is of increasing concern, representing about 75% of total waste.^8^^,^^12^ Leakage of microplastics from landfills or during recycling, along with their wide-spread presence in drinking water, air, animal, and human tissue, has been linked to direct harm to human health, including digestive health.^70–72^

A prerequisite for developing interventions is viewing current waste disposal practices. A recent document on appropriate waste disposal in endoscopy provides guidance on conducting waste audits and suggests possible implementation approaches.^73^

Appropriate waste disposal not only benefits the environment but also reduces costs.^67^ Hazardous waste requires either autoclaving or incineration, both of which are costly. Its disposal is more than 3 times as expensive as landfill waste.^67^ Autoclaving and incineration also consume energy, whereas incineration releases toxic chemicals and greenhouse gases at levels far greater than recycling or landfill.^74^^,^^75^ Regulations for medical waste vary internationally. For example, most medical waste in Europe requires incineration. Among disposal practices, proper segregation of hazardous waste likely offers the greatest environmental benefit by reducing both emissions and toxic byproducts. Targeted interventions can lower hazardous waste volumes by at least 41%.^76^ Misclassification of items such as nonsharp instruments (eg, polypectomy snares) or nonbloody suction containers contributes significantly to misplaced hazardous waste.^73^

Recycling opportunities depend on local regulations and institutional contracts. In 1 study, the introduction of a recycling program reduced waste by 7.5%.^77^ Packaging materials such as cardboard and labeled plastics are often the most feasible to recycle. However, poor labeling and limited awareness of recyclable materials remain barriers. Moreover, the overall benefit of plastic recycling has been increasingly questioned, as more than 95% of plastic waste is still discarded after a single use.^78^

### Travel

9. *Perform telehealth visits when feasible, clinically appropriate, and preferred by the patient.*

Travel is an inherent aspect of endoscopy, whether for the procedure itself, preprocedure consultation, or follow-up care. Because most patients travel by car, this significantly contributes to emissions, averaging 0.17 kgCO_2_e/km (0.27 kgCO_2_e/mile).^79^^,^^80^ Patients and staff travel together may account for a third to half of the total emissions associated with endoscopy.^10–12^

Telehealth or virtual visits can substantially reduce the environmental impact of health care.^81–83^ In the post-COVID-19 era, virtual visits have been widely accepted by patients and practitioners and appear safe and effective.^81^^,^^84^ Phone consultations have also improved attendance and resulted in high patient satisfaction.^85^^,^^86^ Routine follow-up visits and postendoscopy review of normal biopsy results can often be managed virtually, as is already common practice in many units. However, low reimbursement remains a major barrier to broader adoption of telehealth.

10. *Maximize double procedures on the same days for patients requiring more than 1 procedure.*

Patient travel can also be reduced by combining procedures on the same day―so-called “double procedures” (eg, colonoscopy and EGD (upper endoscopy), or EGD and capsule endoscopy). Appointments may also be coordinated with other clinical services, such as laboratory tests or radiology. Although decreased reimbursement for same-day procedures may be a barrier, such practices not only reduce travel but also eliminate the need for additional sedation, lower procedural risk, and reduce the burden of taking an extra day off.

A review of current practices can help identify which visits are suitable for virtual care and where double procedures may be scheduled. On the basis of the benchmark, the endoscopy team can set individual goals for practice change (eg, a 50% increase over 1 year). Patient acceptance and satisfaction may also be assessed.

### Energy

11. *Measure the endoscopy unit’s energy use.*

Medical facilities require large amounts of energy, often consuming more than twice as much per unit of surface area as other commercial buildings.^87^ HVAC accounts for approximately 45% of all energy needs, whereas lighting, computers, and office equipment (eg, printers) make up only 15%.^87^ A U.S. study reported that HVAC and energy use in endoscopy represented 45% of all emissions,^11^ and a study from France found that energy use accounted for 12% of emissions.^12^ Other studies have varied in their methods of assessing energy needs, making comparisons difficult.^9^^,^^10^^,^^68^

Although hospitals operate continuously, endoscopy units are used intermittently, offering opportunities to save energy when spaces are not in use. A review of the energy use patterns may identify potential efficiencies. Energy audits can be conducted with facility management, engineering departments, or external agencies. Environmental impacts will also depend on the local energy mix, that is, fossil fuel versus renewable sources, which vary by institution and region. Energy use can then be converted into carbon emissions per unit or per procedure.

12. *Reduce the endoscopy unit’s energy use.*

Endoscopy units should also be made more efficient wherever possible. Potential interventions include switching to light-emitting diode lighting,^87–90^ which consumes 90% less energy than traditional bulbs and lasts up to 25 times longer.^88^ Motion-activated lighting can further reduce energy use from lighting by as much as 50%.^89^^,^^90^ Additional practices include powering down or turning off electrical de-vices.^91^ Notably, devices in sleep mode still consume 1 to 5 W of power. Turning off computers, printers, and scanners when not in use can reduce energy use by 60% to 90%.^92^

Energy use can further be reduced by optimizing the use of endoscope washers (eg, placing 2 endoscopes in a 2-tray washer). Automated washers consume about 0.3 kWh per procedure, and reprocessing accounts for roughly 5% of carbon emissions.^11^^,^^68^

Potential HVAC changes may be explored with building engineers. Depending on climate zone and season, adjusting temperature setpoints may save energy (eg, higher in summer and lower in winter).^87^ Reducing air exchange rates to the minimum required, and pausing or further lowering exchange rates when rooms are not in use, can also decrease energy demand. Given the large contribution of HVAC to overall emissions in endoscopy, units should aim to maximize the efficient use of available space, thereby reducing emissions per procedure.

## Defining Success

In an ideal scenario, an endoscopy unit might implement all recommended actions. Realistically, however, the transition to sustainable practice will take time. Individual units may need to focus first on actions that are most feasible. For example, a unit without the resources to appoint a sustainability lead or establish a green team may still be able to initiate changes in waste management, increase the use of reusables, and improve the appropriateness of procedures. In higher-resourced settings, more ambitious steps, such as conducting energy or waste audits, may be both feasible and achievable.

To account for the diverse resources and starting points of endoscopy units, outcomes for each indicator are defined as targets or aspirational goals, depending on the number of actions achieved. The proposed actions and targets may be refined and modified over time as evidence grows and as we learn more about which areas of endoscopy have the greatest environmental impact.

## Barriers and Possible Solutions

Several potential barriers may hinder the implementation of sustainable practices. First, cost is a common concern. Although most proposed actions should not incur additional expense, some will require additional staff time and resources. For example, the time devoted to a sustainability lead role will vary between units and should be defined as part of the sustainability plan. Engaging building engineers and implementing HVAC or energy efficiency changes may involve upfront costs; however, investments in reducing energy needs can result in long-term savings.^85^^,^^86^

Second, leadership support is essential. Early engagement―such as dedicated presentations and discussion of financial implications―can secure buy-in. Challenges related to reimbursement, for example, reduced remuneration for telemedicine, should also be addressed.

Third, there may be concerns that sustainability efforts could reduce procedural efficiency. In general, clinicians are more likely to adopt alternative practices when they are evidence-based and feasible in daily workflow. Optimized utilization can not only lower the environmental impact per procedure but can also improve unit efficiency and translate into cost savings.

Finally, concerns about infection prevention may hinder adoption of sustainable practices, even in the absence of supporting evidence (eg, use of sterile water).^93^ These barriers can be mitigated by involving local representatives from infection control and quality assurance. Partnership should include discussion of both the advantages and disadvantages of potential practice changes, beyond environmental benefits, such as cost savings or indirect health impacts from reusable instruments.^60–62^^,^^93^

## Conclusion and Future Directions

The outlined indicators provide guidance for implementing sustainable practices in the endoscopy unit. Adopting these practices can reduce the environmental impact of GI endoscopy while maintaining high-quality care. Although the precise environmental benefits of each indicator are not yet known, their value lies in achieving both absolute reductions in carbon emissions and waste, as well as fostering cultural change. Even small adjustments in daily practice may inspire broader shifts in mindset.

At the same time, efforts should be directed toward engaging the industry to redesign instruments and supplies according to the principles of a circular economy. Implementation of sustainable practices may also be supported by advances in information technology and automated systems that monitor and optimize the energy use of equipment, HVAC, and lighting.^51^ Similarly, electronic medical record systems could be designed to incorporate and propose environmentally preferable choices for equipment or clinical pathways. From a professional society perspective, guidelines should increasingly consider sustainability as part of clinical decision-making.

Finally, there is also a significant opportunity for research. Future work may, for instance, include cost and environmental benefit analyses of implementing sustainability indicators.

Ultimately, the proposed indicators and associated practice changes should be embedded in a broader framework that enables guidance, monitoring, and recognition of endoscopy units. This document provides a foundation for developing such a program and integrating it into existing or new endoscopy unit recognition programs. Conversations are underway with national and global leaders regarding the dissemination, promotion, and implementation of these programs. We hope that the proposed indicators will be adapted and customized for use worldwide.

## Summary

The intent of this document is to guide endoscopy units to transition toward a sustainable practice and reduce the impact of GI endoscopy on the environment while maintaining quality of care. It lists 12 sustainability indicators within 7 categories of clinical practice and pro-poses actions for each indicator.

## Supplementary Material

gwag030_Supplementary_Data
